# Phase I pharmacokinetic, safety, and preliminary efficacy study of tiragolumab in combination with atezolizumab in Chinese patients with advanced solid tumors

**DOI:** 10.1007/s00280-024-04650-y

**Published:** 2024-03-07

**Authors:** Colby S. Shemesh, Yongsheng Wang, Andrew An, Hao Ding, Phyllis Chan, Qi Liu, Yih-Wen Chen, Benjamin Wu, Qiong Wu, Xian Wang

**Affiliations:** 1 Clinical Pharmacology, Genentech Inc., South San Francisco, CA USA; 2 Clinical Trial Center, West China Hospital, Sichuan University, Chengdu, China; 3 Safety Science, F. Hoffmann-La Roche Ltd, Beijing, China; 4 Bioanalytical Science, Genentech Inc., South San Francisco, CA USA; 5 Product Development Oncology, F. Hoffmann-La Roche Ltd, Shanghai, China; 6 Sir Run Run Shaw Hospital Zhejiang University School of Medicine, Hangzhou, China

**Keywords:** Combination anti-TIGIT and anti-PD-L1 cancer immunotherapy, Phase I clinical trials, Immune check point inhibitors, Advanced solid tumors, Ethnic sensitivity and population bridging

## Abstract

**Purpose:**

Tiragolumab is an immunoglobulin G1 monoclonal antibody targeting the immune checkpoint T cell immunoreceptor with immunoglobulin and immunoreceptor ITIM domains. Targeting multiple immune pathways may improve anti-tumor responses. The phase I YP42514 study assessed the pharmacokinetics (PK), safety, and preliminary efficacy of tiragolumab plus atezolizumab in Chinese patients with advanced solid tumors.

**Methods:**

Adult patients from mainland China with Eastern Cooperative Oncology Group performance score 0/1, life expectancy of ≥ 12 weeks, and adequate hematologic/end organ function were eligible. Patients received tiragolumab 600 mg and atezolizumab 1200 mg intravenous every 3 weeks. Key endpoints were PK (serum concentrations of tiragolumab and atezolizumab) and safety. Results from this study were compared with the global phase I study, GO30103 (NCT02794571).

**Results:**

In this study, 20 patients received a median of five doses of tiragolumab plus atezolizumab. Median age was 57.5 years, 85.0% of patients were male and the most common tumor type was non-small cell lung cancer. Exposures in Chinese patients were comparable to the global GO30103 population: geometric mean ratio was 1.07 for Cycle 1 tiragolumab area under the concentration–time curve_0–21_ and 0.92 and 0.93 for Cycle 1 peak and trough atezolizumab exposure, respectively. Treatment-related adverse events were consistent across the Chinese and global populations. Two patients (10.0%) in this study achieved a partial response.

**Conclusion:**

In this study, tiragolumab plus atezolizumab was tolerable and demonstrated preliminary anti-tumor activity. There were no meaningful differences in the PK or safety of tiragolumab plus atezolizumab between the Chinese and global populations.

**Clinical trial registration number**: China Clinical Trial Registry Identifier CTR20210219/YP42514. Date of registration 16 March 2021.

**Supplementary Information:**

The online version contains supplementary material available at 10.1007/s00280-024-04650-y.

## Introduction

Malignant tumors are consistently among the top five causes of death in China, with rates increasing since 2000 [1]. In 2022, there were approximately 4,820,000 new cancer cases and 3,210,000 cancer deaths in China, with lung cancer being the leading cause of cancer death [2]. In 2012, new cases of cancer diagnosed in patients aged 65 years and older represented 47.5% of the total number of new cancers worldwide, with 1.5 million cases in China alone (approximately one-fifth of the worldwide total) [3]. More efforts are needed to deliver effective cancer care and support healthy aging in China [2].

To address these unmet medical needs and encourage innovative drug development, the drug regulatory landscape in China has changed in recent years, resulting in a substantial increase in phase I clinical trials conducted in China [4–6]. Ethnic sensitivity analysis early in drug development is encouraged by Chinese regulatory bodies to bridge the gap between global clinical data and Chinese populations. Conducting comprehensive assessments in Chinese populations may enable shorter regulatory review and development of early global studies, hopefully leading to more innovative drug approvals and improved patient outcomes [4].

Cancer immunotherapies, such as immune checkpoint inhibitors (ICI), have greatly advanced cancer therapy options in recent years [7]. However, their clinical use is limited by challenges associated with variable patient response rates, drug resistance, tumor heterogeneity, changing expression of targeted receptors/checkpoints and immune-mediated damage of normal tissues [7]. Tumor-induced immunosuppression is mediated by multiple pathways; therefore, development of effective combination regimens may provide the answer to inducing complete or durable anti-tumor responses [8].

The T cell immunoreceptor with immunoglobulin and immunoreceptor ITIM domains (TIGIT) is an inhibitory immune checkpoint present on activated T cells and natural killer (NK) cells in multiple cancers [9, 10]. TIGIT binds with high affinity to the poliovirus receptor (PVR) and is associated with impaired T cell and NK cell function, as well as impaired anti-tumor immunity [9, 10]. In several human tumor types, TIGIT and programmed cell death protein-1 (PD-1) are co-expressed by tumor antigen-specific T cells and tumor-infiltrating lymphocytes (TILs) [9, 10]. Preventing TIGIT signaling through the use of anti-TIGIT ICIs may restore immune responses in tumor cells [9, 11].

Tiragolumab is a first-in-class human immunoglobulin (Ig) G1/kappa monoclonal antibody (mAb) targeting TIGIT, with an intact Fc region that blocks interaction of TIGIT with PVR [12]. Inhibition of both the TIGIT and PD-1/programmed death-ligand 1 (PD-L1) pathways may improve anti-tumor responses compared with monotherapy [10, 11]. In mouse tumor models, simultaneous inhibition of the TIGIT/PVR and PD-1/PD-L1 pathways improved anti-tumor activity compared with blockade of either pathway alone [10]. In patients with non-small cell lung cancer (NSCLC) [10] and melanoma [13], inhibition of both pathways increased in vitro proliferation, cytokine production, and anti-tumor function of CD8 + TILs.

Safety and efficacy findings of tiragolumab in combination with the PD-L1 inhibitor atezolizumab in patients with solid tumors have been published from the phase I GO30103 study (NCT02794571) [12, 14], which also had a tiragolumab monotherapy cohort, and the phase II CITYSCAPE study (NCT03563716) [15]. In the GO30103 study, tiragolumab plus atezolizumab demonstrated preliminary anti-tumor activity, with objective responses observed in NSCLC and head and neck squamous cell carcinoma [12]. Tiragolumab plus atezolizumab was well tolerated and showed clinically meaningful improvements in objective response rate (ORR), progression-free survival (PFS), and overall survival (OS) versus atezolizumab alone in the CITYSCAPE study [15].

The pharmacokinetics (PK) of tiragolumab given alone or in combination with atezolizumab have previously been characterized in the secondary objective of the GO30103 study, where 76 out of 200 (38.0%) patients were Asian (outside of Mainland China). Overall, PK data were consistent between patients of Western and Asian origin. Although pharmacogenomics is not expected to play a role in tiragolumab PK, it is important to assess whether the PK and safety profile of tiragolumab is similar between Chinese and Western populations.

This study is the first to report PK, safety, and preliminary anti-tumor activity of tiragolumab plus atezolizumab in Chinese patients with advanced or metastatic solid tumors from a phase I study (YP42514, CTR20210219). This study also reports PK and safety data from the global GO30103 study for comparison with the Chinese population in this study [12, 14].

## Materials and methods

### Patients

Eligible patients were residents in mainland China and were aged 18 years or older with locally advanced or metastatic solid tumors, an Eastern Cooperative Oncology Group performance score (ECOG PS) of 0 or 1, a life expectancy of at least 12 weeks, and adequate hematologic and end organ function. Patients were excluded if they met any of the following general exclusion criteria: pregnancy, lactation, breastfeeding or having the intention of becoming pregnant during study treatment or within 5 months after the final dose of study treatment; treatment with investigational therapy within 28 days prior to initiation of study treatment; known clinically significant liver disease; significant cardiovascular disease within 3 months prior to Day 1 of Cycle 1, unstable arrhythmia, or unstable angina; poorly controlled Type 2 diabetes mellitus defined as a screening hemoglobin A_1c_ of ≥ 8%, or fasting plasma glucose ≥ 160 mg/dL (or 8.8 mmol/L). Inclusion and exclusion criteria are detailed in Online resource 1.

### Study design

YP42514 is an ongoing, phase I, open-label study of tiragolumab in combination with atezolizumab in Chinese patients with locally advanced or metastatic solid tumors. Enrolled patients received tiragolumab (600 mg) plus atezolizumab (1200 mg) by intravenous infusion on the first day of each 21-day cycle, until unacceptable toxicity and/or loss of clinical benefit. Treatment with atezolizumab beyond disease progression was permitted in the absence of unacceptable toxicity (full criteria regarding treatment beyond progression are detailed in Online resource 2). Dose reductions for tiragolumab or atezolizumab were not permitted in this study. Initial and subsequent doses of tiragolumab were administered prior to atezolizumab infusions, with an intervening observation period.

The study protocol and protocol amendments were approved by the institutional review board or ethics committee and complied with Good Clinical Practice guidelines, the principles of the Declaration of Helsinki, and applicable laws and regulations. All patients provided written informed consent.

### Endpoints and assessments

The primary endpoints of this study were serum concentrations of tiragolumab and atezolizumab (µg/mL) at specified timepoints. The safety of tiragolumab in combination with atezolizumab was also a key endpoint in this study. Secondary endpoints included immunogenicity and efficacy endpoints.

Samples for determination of tiragolumab and atezolizumab PK were obtained in the first dosing cycle on Days 1, 2, 8, and 15, and on the first day of any subsequent cycles, followed by sparse peak and trough serum collection. Estimated PK parameters included minimum serum concentration (C_min_), maximum plasma concentration (C_max_), accumulation ratio based on concentrations after the first dose and at steady state, area under the concentration–time curve (AUC) from Day 0–21, AUC from Day 0 extrapolated to infinity, systemic clearance, volume of distribution at steady-state, and terminal half-life. Tiragolumab and atezolizumab serum concentrations were measured using validated enzyme-linked immunosorbent assays (ELISA). The serum concentration of tiragolumab was determined with a validated ELISA with a lower limit of quantification of 25 ng/mL using two conjugated reagents to capture tiragolumab: biotin-conjugated TIGIT-flag and digoxigenin (DIG)-conjugated TIGIT-flag. The serum concentration of atezolizumab was determined with a validated ELISA with a lower limit of quantification of 60 ng/mL using humanized PD-L1 Fc as capture and biotinylated anti-framework antibody as detection [16].

Safety endpoints included frequency and severity of adverse events (AEs), with the severity of AEs being determined according to National Cancer Institute Common Terminology Criteria for Adverse Events version 5.0 (NCI CTCAE v5.0), and the severity of cytokine release syndrome (CRS) determined according to the American Society for Transplantation and Cellular Therapy (ASTCT) consensus grading scale [17]. Immunogenicity endpoints included the prevalence of anti-drug antibodies (ADAs) to tiragolumab at baseline, and the incidence of ADAs to tiragolumab during the study. ADAs against tiragolumab in human serum were detected at multiple time points before, during, and after treatment with tiragolumab using a validated screening and confirmatory assay with two conjugated reagents to capture: biotin-conjugated tiragolumab and DIG-conjugated tiragolumab. Using a surrogate positive control, the ADA method had a relative sensitivity of 2.9 ng/mL and was able to detect 100 ng/mL of surrogate positive control in the presence of up to 250 μg/mL of tiragolumab.

Efficacy endpoints included confirmed ORR and duration of response (DOR). Confirmed ORR was defined as the proportion of patients who had a confirmed objective response of complete response or partial response according to Response Evaluation Criteria in Solid Tumors, version 1.1 (RECIST v1.1) on two consecutive occasions at least 4 weeks apart, as determined by the investigator (confirmation was not required for patients with malignant lymphoma). DOR was defined as the time from the date of the first occurrence of a documented objective response until the first date of disease progression or death, whichever occurred first for patients with a confirmed response. Kaplan–Meier methodology was used to estimate the median DOR. Tumor assessments were performed at baseline and every 6 weeks for 48 weeks from Day 1 Cycle 1, and then every 9 weeks thereafter regardless of treatment delays, until radiographic disease progression per RECIST v1.1 or loss of clinical benefit, withdrawal of consent, death, or study termination by the Sponsor, whichever occurred first.

### Sample size and statistical analysis

There was a target enrollment of 20 patients, with at least 12 PK-evaluable patients to provide sufficient data to characterize the PK and safety of tiragolumab in combination with atezolizumab. There was no formal hypothesis testing for this study: PK parameters were summarized using descriptive statistics. The tiragolumab and atezolizumab PK-evaluable population included all patients who received at least one dose of the respective study treatment and had at least one corresponding post-baseline PK sample available. The safety population included all patients who received any amount of study treatment. Immunogenicity analyses included patients with any tiragolumab ADA assessments.

### Comparison with global GO30103 cohort

To support the dosing approach, findings from this current study were compared with results from a cohort of the global GO30103 study, where patients from Australia, Canada, France, Korea, Spain, and the United States received the same combination and dosing regimen [12, 14]. GO30103 was a multicenter, open-label, dose-escalation and expansion phase Ia/Ib study of tiragolumab alone (2–1200 mg, phase Ia) or in combination with atezolizumab (1200 mg, phase Ib) in patients with advanced solid tumors for whom standard treatment did not exist or was ineffective [12, 14]. Samples for determination of tiragolumab and atezolizumab PK were collected at multiple timepoints before and after dosing on Day 1, 2, 8, and 15 of Cycle 1, Day 1 of Cycles 2, 3, 4, and 8, and then every 8 cycles thereafter [12, 14]. Serum samples for the detection of ADAs were collected pre-dose on Day 1 of Cycles 1, 2, 3, 4, and 8, and assessed using a validated immunoassay [14]. The comparison between the two studies is for descriptive purposes only: no formal statistical comparison of the data sets was completed.

## Results

From a total of 25 patients screened, 20 patients were enrolled into the YP42514 study from two centers in China by the data cut-off date (February 10, 2022). A CONSORT diagram is included in Online resource 3. Patients received a median of five doses of tiragolumab (600 mg) plus atezolizumab (1200 mg). Baseline characteristics and demographics of the 20 patients included in this study, compared with those from one cohort of the global GO30103 study, [12, 14] are shown in Table 1. The Chinese population from the YP42514 study had a median age of 57.5 years (range 44–73) and was predominantly male (85.0%). Among the previously treated patients, 50.0% had received two or more lines of prior anti-cancer therapy. The most common tumor type was NSCLC (55.0%). All but one patient had stage IV disease at enrollment.

**Table 1:** Baseline characteristics and patient demographics in YP42514 and GO30103

Characteristics		YP42514 (*N* = 20)	GO30103 (*N* = 108)^a^
Age, years	Median (range)	57.5 (44–73)	61.0 (25–82)
Weight, kg	Median (range)	63.7 (45.3–73.8)	65.0 (41.0–114.9)
Sex, *n* (%)	Male	17 (85.0)	58 (53.7)
	Female	3 (15.0)	50 (46.3)
Race, *n* (%)	Asian	20 (100)	32 (29.6)
	Black or African American	0	0
	White	0	42 (38.9)
	Unknown	0	34 (31.5)
Ethnicity, *n* (%)	Hispanic or Latino	0	5 (4.6)
	Not Hispanic or Latino	20 (100)	69 (63.9)
	Not stated	0	29 (26.9)
	Unknown	0	5 (4.6)
Tobacco use history, *n* (%)	Never	8 (40.0)	–
	Current	1 (5.0)	–
	Former	11 (55.0)	–
ECOG PS (at baseline), *n* (%)	0	9 (45.0)	34 (31.5)
	1	11 (55.0)	72 (66.7)
	2	0	2 (1.9)
Primary cancer history type, *n* (%)	Bile duct cancer	1 (5.0)	1 (0.9)
	Colorectal cancer	2 (10.0)	8 (7.4)
	Esophageal cancer	0	20 (18.5)
	Gastric cancer	2 (10.0)	6 (5.6)
	Melanoma	1 (5.0)	3 (2.8)
	NSCLC	11 (55.0)	16 (14.8)
	Uterine cancer	1 (5.0)	1 (0.9)
	Other^b^	2 (10.0)	53 (49.1)
Staging (AJCC), *n* (%)	IIIA	1 (5.0)	–
	IV	19 (95.0)	–
Line of latest prior cancer therapy, *n* (%)	1st	8 (42.1)	25 (23.1)
	2nd	2 (10.5)	31 (28.7)
	3rd	4 (21.1)	18 (16.7)
	4th	4 (21.1)	19 (17.6)
	5th–9th	0	15 (13.8)
	No prior treatment	1 (5.3)	0
Received prior immunotherapy, *n* (%)		6 (30.0)	27 (25.0)

*AJCC* American Joint Committee on Cancer, *ECOG PS* Eastern Cooperative Oncology Group performance status, *NSCLC* non-small cell lung cancer, *Q3W* every 3 weeks

^a^Phase Ib tiragolumab 600 mg Q3W plus atezolizumab 1200 mg Q3W cohort; ^b^The most common tumor type has been listed for the GO30103 study. Other tumor types in YP42514: small intestine (*n* = 1) and rectal (*n* = 1). Other tumor types in GO30103: anal (*n* = 1), bladder (*n* = 3), breast (*n* = 9), cervical (*n* = 5), gastro-esophageal junction (*n* = 7), head and neck (*n* = 9), kidney (*n* = 9), small cell lung (*n* = 1), and ovarian (*n* = 9)

In the global GO30103 study, at data cut-off (October 1, 2021), 108 patients had received a median of four doses of tiragolumab (600 mg) plus atezolizumab (1200 mg). Patient demographics in the global GO30103 study were similar to those in the Chinese population in the YP42514 study. However, there were some key differences: in the GO30103 cohort, the median age (61.0 years [range 25–82]) was higher and fewer patients were male (53.7%) compared with YP42514, and the most common tumor type was esophageal cancer (18.5%; Table 1). There was a numerically lower proportion of patients with baseline ECOG PS of 1 in the YP42514 study (55.0%) compared with the global GO30103 cohort (66.7%). The proportion of patients who had received prior immunotherapy was generally similar between studies (30.0% in the YP42514 study and 25.0% in the global GO30103 cohort).

At data cut-off in both the YP42514 and GO30103 studies, most patients (70.0% and 89.9%, respectively) had discontinued study treatment, with the most common reason for treatment discontinuation being disease progression (55.0% and 72.2%, respectively).

### Treatment exposure

Treatment exposure was similar in the Chinese population from the YP42514 study compared with the respective global GO30103 cohort. The median treatment duration for tiragolumab plus atezolizumab in the YP42514 study was 2.8 months (range 0–8.3 months), with 55.0% of patients receiving up to 3 months of treatment. Similarly, the median treatment duration for tiragolumab plus atezolizumab in the global GO30103 cohort was 2.7 months (range 0–45 months), with 53.7% of patients receiving up to 3 months of treatment. In both the YP42514 and GO30103 studies, the median dose intensity was 100.0% for both tiragolumab (range 90.3–100% and 99–100%, respectively) and atezolizumab (range 90.3–100% and 98–100%, respectively).

### Pharmacokinetics

**Fig. 1:**
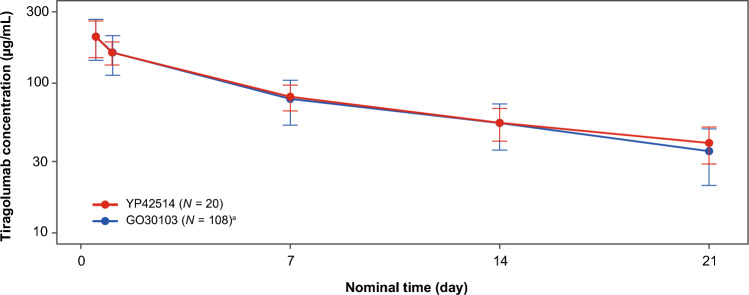
Plot of Cycle 1 serum tiragolumab concentrations (µg/mL; mean ± SD). ^a^Global GO30103 study, phase Ib tiragolumab 600 mg Q3W plus atezolizumab 1200 mg Q3W cohort. *Q3W* every 3 weeks, *SD* standard deviation

**Fig. 2:**
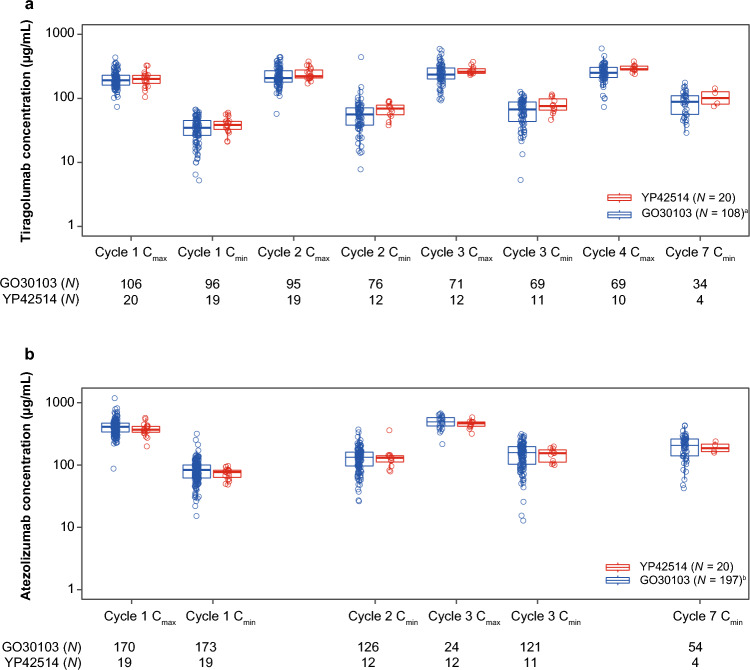
Boxplot of Cycles 1–7 serum tiragolumab and atezolizumab concentrations (µg/mL). ^a^Global GO30103 study, phase 1b tiragolumab 600 mg Q3W plus atezolizumab 1200 mg Q3W cohort; ^b^Atezolizumab PK data have been pooled from all patients in GO30103 who had serum atezolizumab concentration levels after administration of tiragolumab 2–1200 mg Q3W in combination with atezolizumab 1200 mg Q3W. *C*_*max*_ maximum serum concentration, *C*_*min*_ minimal serum concentration, *PK* pharmacokinetics, *Q3W* every 3 weeks

In the YP42514 study, all patients had serum tiragolumab PK-evaluable samples after administration of tiragolumab plus atezolizumab. The serum PK profile of tiragolumab appeared biphasic with a rapid distribution followed by a slower elimination phase, as shown in Fig. 1. Tiragolumab concentrations increased over time, ranging from a geometric mean (% coefficient of variation [CV]) C_max_ of 209 μg/mL (22.6%) at Cycle 1 to 293 μg/mL (13.2%) at Cycle 4. For C_min_, values were 37.4 μg/mL (30.4%) at Cycle 1 and 77.5 μg/mL (29.1%) at Cycle 3 (Tables 2 and 3; Fig. 2).

**Table 2:** Summary statistics for Cycle 1 tiragolumab PK parameters

PK parameter^a^	YP42514		GO30103 [14]^b^		GMR (90% CI)
	*N*	GM (%CV)	*N*	GM (%CV)	
C_max_ (µg/mL)	18	209 (22.6)	99	198 (30.1)	1.06 (0.95–1.17)
AUC_0–inf_ (µg*day/mL)	8	1910 (22.4)	45	1830 (33.5)	1.04 (0.88–1.23)
AUC_0–21_ (µg*day/mL)	18	1640 (20.3)	95	1530 (29.9)	1.07 (0.98–1.18)
C_min_ (µg/mL)	17	37.4 (30.4)	89	31.2 (55.2)	1.20 (1.03–1.40)
t_1/2_ (day)	8	9.73 (28.9)	45	9.52 (19.1)	1.02 (0.84–1.24)
CL (L/day)	8	0.314 (22.4)	45	0.324 (30.2)	0.969 (0.83–1.14)
V_ss_ (L)	8	4.18 (22.7)	45	4.29 (29.8)	0.974 (0.83–1.15)

*%CV* coefficient of variation, *AUC*_*0–21*_ area under Cycle 1 (21 days) serum concentration curve, *AUC*_*0–inf*_ area under serum concentration curve from time zero extrapolated to infinity, *CI* confidence interval, *CL* total body clearance, *C*_*max*_ maximum serum concentration, *C*_*min*_ minimal serum concentration, *GM* geometric mean, *GMR* geometric mean ratio, *PK* pharmacokinetic, *Q3W* every 3 weeks, *t*_*1/2*_ half-life, *V*_*ss*_ volume of distribution at steady state

^a^Given limited sampling at the terminal phase and large extrapolation from AUC_0–21_ versus AUC_0–inf_, t_1/2_, AUC_0–inf_, CL, and V_ss_ estimates are considered to be approximate; ^b^Global GO30103 study phase Ib tiragolumab 600 mg Q3W plus atezolizumab 1200 mg Q3W cohort

**Table 3:** Summary statistics for Cycles 1–7 tiragolumab (600 mg) and atezolizumab (1200 mg) concentrations (µg/mL) following IV administration Q3W

Visit	YP42514		GO30103 [14]		GMR (90% CI)
	*N*	GM (%CV)	*N*	GM (%CV)	
Tiragolumab^a^					
Cycle 1 C_max_	20	197 (28.4)	106	196 (30.1)	1.01 (0.89–1.13)
Cycle 1 C_min_	19	38.4 (29.9)	96	31.6 (54.4)	1.22 (1.05–1.40)
Cycle 2 C_max_	19	241 (21.7)	95	217 (34.8)	1.11 (1.00–1.23)
Cycle 2 C_min_	12	65.1 (28.9)	76	51.0 (63.5)	1.28 (1.07–1.53)
Cycle 3 C_max_	12	274 (14.4)	71	238 (39.3)	1.15 (1.04–1.28)
Cycle 3 C_min_	11	77.5 (29.1)	69	57.9 (60.8)	1.34 (1.11–1.61)
Cycle 4 C_max_	10	293 (13.2)	69	246 (35.1)	1.19 (1.08–1.32)
Cycle 7 C_min_	4	102 (31.2)	34	81.7 (46.9)	1.25 (0.88–1.78)
Atezolizumab^b^					
Cycle 1 C_max_	19	369 (24.3)	170	401 (29.3)	0.920 (0.83–1.02)
Cycle 1 C_min_	19	72.4 (21.1)	173	78.0 (45.0)	0.928 (0.84–1.02)
Cycle 2 C_min_	12	129 (40.1)	126	121 (49.0)	1.07 (0.86–1.31)
Cycle 3 C_max_	12	448 (15.8)	24	479 (26.6)	0.935 (0.83–1.05)
Cycle 3 C_min_	11	143 (25.9)	121	137 (57.8)	1.04 (0.89–1.22)
Cycle 7 C_min_	4	190 (19.7)	54	185 (52.9)	1.03 (0.82–1.29)

*%CV* coefficient of variation, *CI* confidence interval, *C*_*max*_ maximum serum concentration, *C*_*min*_ minimal serum concentration, *GM* geometric mean, *GMR* geometric mean ratio, *IV* intravenous, *PK* pharmacokinetic, *Q3W* every 3 weeks

^a^Phase Ib tiragolumab 600 mg Q3W plus atezolizumab 1200 mg Q3W cohort; ^b^Atezolizumab PK data in GO30103 have been pooled from all patients who had serum atezolizumab concentration levels after administration of 2–1200 mg tiragolumab Q3W in combination with 1200 mg atezolizumab Q3W

All patients had serum atezolizumab PK-evaluable samples after administration of tiragolumab plus atezolizumab. Atezolizumab concentrations also increased over time, with a geometric mean (%CV) C_max_ of 369 μg/mL (24.3%) at Cycle 1 and 475 μg/mL (18.1%) at Cycle 4. For C_min_, values were 72.4 μg/mL (21.1%) at Cycle 1 and 143 μg/mL (25.9%) at Cycle 3 (Fig. 2; Table 3). Interpatient variability was low to moderate for both tiragolumab and atezolizumab.

PK parameters in this Chinese population were comparable to the global GO30103 study population [14] (Figs. 1 and 2; Tables 2 and 3), with a geometric mean ratio (GMR) of 1.07 (90% confidence interval [CI]: 0.98–1.18) for Cycle 1 tiragolumab AUC_0–21_ (Table 2), and a GMR of 0.92 (90% CI: 0.83–1.02) and 0.93 (90% CI: 0.84–1.02) for Cycle 1 peak and trough atezolizumab exposure, respectively (Table 3). Peak and trough exposures for tiragolumab plus atezolizumab within Cycles 1–7 are summarized in Table 3.

### Efficacy

The confirmed ORR with tiragolumab plus atezolizumab in the YP42514 study was 10.0%, with both patients achieving a partial response. The median DOR was non-estimable at the cut-off date, as the two patients with a response were still in follow-up and had not progressed or died; the individual DORs of the two responders were 3 and 6 months (censored). Seven patients (35.0%) achieved a best response of stable disease. The disease control rate was 45.0%.

### Immunogenicity

Among tiragolumab ADA-evaluable patients, none tested positive for tiragolumab ADAs at baseline or post-baseline in the YP42514 study. In the phase Ia portion of the global GO30103 study [14], no treatment-emergent tiragolumab ADAs had been detected among 37 evaluable patients. In the phase Ib portion of the GO30103 study, one patient in the tiragolumab 600 mg cohort had baseline tiragolumab ADAs (0.5% prevalence) [14]. Post-baseline, four out of 207 evaluable patients (1.9%) were positive for treatment-emergent tiragolumab ADAs: two patients were from the tiragolumab 400 mg dose group and the other two patients were from the 600 mg tiragolumab dose group [14].

### Safety

Overall, treatment with tiragolumab plus atezolizumab was well tolerated in this Chinese population and the observed safety profile was consistent with that previously reported for this combination in the GO30103 study [12], as shown in Table 4. Frequency of treatment-related AEs, all-cause grade 3/4 AEs, and serious AEs and AEs leading to withdrawal from study treatment were generally similar for this Chinese population (85.0%, 40.0%, 35.0%, and 5.0%, respectively) and patients in the global GO30103 study (71.3%, 41.7%, 28.7%, and 5.6%, respectively) [12]. No grade 5 AEs were reported in the YP42514 study.

**Table 4:** Safety summary for patients treated with IV administration of tiragolumab (600 mg) plus atezolizumab (1200 mg) Q3W

Number of patients with ≥ 1 event, *n* (%)	YP42514 (*N* = 20)	GO30103 (*N* = 108)^a^
All-grade AEs	20 (100)	101 (93.5)
Any-grade TRAEs	17 (85.0)	77 (71.3)
Grade 3–4 AEs	8 (40.0)	45 (41.7)
Serious AEs	7 (35.0)	31 (28.7)
Grade 5 AEs	0	5 (4.6)^b^
AEs leading to withdrawal	1 (5.0)	6 (5.6)
AEs leading to dose interruption/modification	1 (5.0)	28 (25.9)

*AE* adverse event, *IV* intravenous, *Q3W* every 3 weeks, *TRAE* treatment-related adverse event

^a^Phase Ib tiragolumab 600 mg Q3W plus atezolizumab 1200 mg Q3W cohort; ^b^None of these events were considered related to any study treatment by the investigator

The most frequently reported AEs for the YP42514 study were increased aspartate aminotransferase (40.0%), rash (40.0%), decreased lymphocyte count (35.0%), pruritus (35.0%), and increased alanine aminotransferase (30.0%); further details of AEs can be found in Online resource 4. Most patients (80.0%) experienced adverse events of special interest (AESIs), with the most common being low-grade immune-mediated hepatitis (lab abnormality 60.0%) and rash (40.0%). AESIs were generally manageable, with few patients requiring treatment interruption or withdrawal (Online resource 5). Lymphopenia, a theoretical risk for tiragolumab, was observed in 40.0% of patients, with most events being low in grade. No events of CRS were reported.

Infusion-related reactions (IRRs) are known side effects of intravenously administered monoclonal antibodies [18]. One out of 20 (5.0%) patients in the YP42514 study experienced an IRR which was a grade 2 non-serious event that occurred within 24 h after the end of infusion (Online resource 5). The patient experienced pyrexia and myalgia which were treated with oxygen therapy and ibuprofen. The pyrexia resolved on the same day, and the IRR event was resolved with no change to the study treatment received. The IRR findings in the YP42514 study were generally consistent with the pattern seen in the phase Ib tiragolumab 600 mg Q3W plus atezolizumab 1200 mg cohort of the global GO30103 study. IRRs were typically rare in this population and occurred in six out of 108 patients (5.6%), which were predominantly low to moderate grade 1–2 events.

AEs related to treatment were experienced by 85.0% of patients, most commonly in the following system organ classes: investigations, 55.0% (most commonly lymphocyte count decreased [25.0%] and aspartate transaminase increased [20.0%]); skin and subcutaneous disorders, 55.0% (most commonly rash [40.0%] and pruritus [35.0%]); and metabolism and nutrition disorders, 30.0%.

One patient discontinued the study treatment due to a grade 3 AE of pneumonitis and another patient had a grade 2 AE of ulcerative colitis, which led to treatment interruption. At the cut-off date, three patients had died; in all three cases, the reported cause of death was progressive disease.

## Discussion

The results of this phase I study showed no meaningful differences in PK characteristics for tiragolumab plus atezolizumab between Chinese and global populations. Data from the global phase I GO30103 study [14] showed that tiragolumab exhibits PK characteristics typical of other IgG1 anti-TIGIT mAbs, including a rapid distribution followed by slower elimination, limited volume of distribution, relatively slow clearance with a long half-life, low to moderate inter-individual variability, and mild accumulation following a once every 3 weeks regimen with linear PK at doses of 100 mg or more [19–21]. The recommended phase II dosing approach for tiragolumab was also generally similar to, and within the range of, approaches for other late-stage mAbs in development targeting the same pathway [19–25].

Treatment with tiragolumab and atezolizumab was well tolerated, and the observed safety profile was consistent with that previously reported for the combination. Frequency of treatment-related AEs, all-cause grade 3/4 AEs, serious AEs, and AEs leading to withdrawal from study treatment were generally similar for this Chinese population and patients in the global GO30103 study [12]. Among tiragolumab ADA-evaluable patients in the YP42514 study, none tested positive for tiragolumab ADAs. This is similar to results from the global GO30103 study, where the ADA incidence rate for tiragolumab was low (less than 2%) [14].

There is a complex interplay of factors that are potentially responsible for interpatient variability in the PK of therapeutic mAbs. As well as mAb clearance, which may be explained by body weight variations between ethnicities, other factors that may differ between ethnicities include differences in lymphatic functions, target expression levels, polymorphisms of the Fc receptor gene, recycling/clearance of IgG, immunogenicity and disease-related factors [26]. There are also large differences in the living environments (including medical availability) between Asian and non-Asian populations [4]. These differences have the potential to alter dose and schedule depending on the therapeutic window of the investigational drug and affect if differences are large enough to be considered clinically meaningful.

Furthermore, ethnic sensitivity is a key factor to consider when aiming to bridge the gap between overseas clinical data and the Chinese population. Investigation in Chinese patients early in drug development is encouraged by the Chinese regulatory body, the National Medicinal Products Administration (NMPA) [4]. The results of ethnic difference analyses could impact the acceptance or approval of a drug, as well as any use or dosage adjustments based on ethnicity [4].

Similar PK and safety profiles of tiragolumab plus atezolizumab between the Chinese population in YP42514 and the global population described in GO30103 are similar to that seen with PD-1 inhibitors. A population pharmacokinetics (PPK) analysis of nivolumab monotherapy, for example, found that nivolumab treatment was not sensitive to race when evaluated in Chinese and non-Asian patients [27]. Also, a systematic review of PPK models of anti-PD-1 mAbs found that race had no clinically meaningful effect on the PK of anti-PD-1 mAbs [28]. In a phase I study in Chinese patients (NCT02825940), anti-PD-L1 mAb atezolizumab was shown to have a safety and PK profile consistent with that previously observed in other non-Chinese patients (Roche data on file).

The harmonization of Chinese regulatory guidance with overseas guidance, an emphasis on approval based on clinical value, and relaxation on imported drug approvals have encouraged the recent rapid development of clinical trials in China [4–6]. This study adds to the growing number of phase I clinical trials developed in mainland China and, to our knowledge, is the first to conduct a comprehensive assessment of tiragolumab in Chinese patients. Only 29.8% of phase I clinical trials in China are conducted in patients with cancer and first-in-Chinese studies account for only 18.0% of clinical trials of newly tested innovative drugs [5]. Despite the acceleration of drug development in China in recent years, more efforts are required to improve the transition of clinical trials of innovative drugs from phase I to phase II, as rates are decreasing [5].

The relatively small sample size is a potential limitation of this YP42514 study, only 20 Chinese patients comprised the PK- and safety-evaluable population. Additionally, the characterization of PK parameters was not statistically powered therefore, there was no formal hypothesis testing for this study.

The dose used in this study (600 mg tiragolumab plus 1200 mg atezolizumab, every 3 weeks) is the same as other studies of tiragolumab, including the phase III studies SKYSCRAPER-01 (NCT04294810; untreated advanced PD-L1-positive NSCLC), SKYSCRAPER-02 (NCT04256421; untreated extensive-stage small cell lung cancer) [29], and SKYSCRAPER-08 (NCT04540211; unresectable advanced esophageal squamous cell carcinoma). SKYSCRAPER-08 enrolled a predominantly Chinese patient population and demonstrated statistically significant and clinically meaningful improvements in PFS and OS for atezolizumab plus tiragolumab in combination with chemotherapy versus chemotherapy alone [30].

In conclusion, the combination of tiragolumab plus atezolizumab demonstrated preliminary anti-tumor activity and was well-tolerated in this Chinese patient population. No meaningful differences in the PK or safety profile of tiragolumab in combination with atezolizumab were observed between this Chinese population and global populations, indicating that dose adjustment by ethnic origin is not required. These results support further investigation of tiragolumab plus atezolizumab combination in Chinese patients with advanced solid tumors.

### Supplementary Information

Below is the link to the electronic supplementary material.

Supplementary file1 (PDF 102 KB)

Supplementary file2 (PDF 72 KB)

Supplementary file3 (PDF 64 KB)

Supplementary file4 (PDF 69 KB)

Supplementary file5 (PDF 26 KB)

## Data Availability

For up-to-date details on Roche's Global Policy on the Sharing of Clinical Information and how to request access to related clinical study documents see here: https://go.roche.com/data_sharing.
