# Supplementary material for: Phase I pharmacokinetic, safety, and preliminary efficacy study of tiragolumab in combination with atezolizumab in Chinese patients with advanced solid tumors
Source: Cancer Chemother Pharmacol. 2024 Mar 7;94(1):45–55. doi: 10.1007/s00280-024-04650-y (PMC11258083; doi:10.1007/s00280-024-04650-y)
Supplement: Supplementary file 1 — Supplementary file1 (PDF 102 KB) [file 280_2024_4650_MOESM1_ESM.pdf]

Title: Phase I pharmacokinetic, safety, and preliminary efficacy study of tiragolumab in combination with atezolizumab in Chinese patients with advanced solid tumors

Authors: Dr. Colby S. Shemesh*, Prof. Yongsheng Wang*, Dr. Andrew An, Ms Hao Ding, Dr. Phyllis Chan, Ms Qi Liu, Dr. Yih-Wen Chen, Dr. Benjamin Wu, Dr. Qiong Wu, Prof. Xian Wang

*Co-first authors

Corresponding author: Colby S. Shemesh, Clinical Pharmacology, Genentech Inc., South San Francisco, CA, USA. E-mail: shemesh.colby@gene.com.

Journal: Cancer Chemotherapy and Pharmacology

Online resource 1 Inclusion and exclusion criteria

Inclusion criteria

- Signed Informed Consent Form
- Age ≥ 18 years at time of signing Informed Consent Form
- Ability to comply with the study protocol
- Eastern Cooperative Oncology Group performance status of 0 or 1
- Life expectancy ≥ 12 weeks
- Adequate hematologic and end organ function, defined by the following laboratory results obtained within 14 days prior to the first study treatment (Day 1 of Cycle 1):
  - Absolute neutrophil count ≥ 1500/μL
  - Lymphocyte count ≥ 500/μL
  - Platelet count ≥ 100,000/μL (without transfusion within 14 days prior to Day 1 of Cycle 1)
  - Hemoglobin ≥ 9 g/dL
    - Patients may be transfused or may receive erythropoietic treatment per local standard-of-care
  - Total bilirubin ≤ 1.5 × upper limit of normal (ULN)
  - Aspartate transaminase (AST), alanine transaminase (ALT) and alkaline phosphatase (ALP) ≤ 2.5 × ULN with the following exception:
    - Patients with documented liver or bone metastases: ALP ≤ 5 × ULN
  - Albumin ≤ 25 g/L (≤ 2.5 g/dL)
- Negative serum pregnancy test for women of childbearing potential (including women who have had a tubal ligation)
    - The pregnancy test must be performed and documented as negative within 14 days prior to Day 1 of Cycle 1
- Current resident of mainland China
- For women of childbearing potential: agreement to remain abstinent (refrain from heterosexual intercourse) or use contraception

- For men: agreement to remain abstinent (refrain from heterosexual intercourse) or use a condom, and agreement to refrain from donating sperm

Exclusion criteria

General exclusion criteria

- Pregnancy, lactation, or breastfeeding
- Significant cardiovascular disease (such as New York Heart Association Class II or greater cardiac disease or myocardial infarction) within 3 months, unstable arrhythmia, or unstable angina
- Known clinically significant liver disease, including active viral, alcoholic, or other hepatitis, cirrhosis, inherited liver disease, or current alcohol abuse
- Poorly controlled Type 2 diabetes mellitus defined as a screening hemoglobin A_{1c} ≥ 8% or a fasting plasma glucose ≥ 160 mg/dL (or 8.8 mmol/L)
- Major surgical procedure within 28 days prior to Day 1 of Cycle 1, or anticipation of need for a major surgical procedure during the study
- Any other disease, metabolic dysfunction, physical examination finding, and/or clinical laboratory finding giving reasonable suspicion of a disease or condition that contraindicates the use of an investigational drug, may affect the interpretation of the results, or may render the patient at high risk from treatment complications

Cancer-specific exclusion criteria

- Any anti-cancer therapy, whether investigational or approved, including chemotherapy, hormonal therapy, and/or radiotherapy, within 3 weeks prior to initiation of study treatment, with the following exceptions:
  - Hormonal therapy with gonadotropin-releasing hormone (GnRH) agonists or antagonists for prostate cancer
  - Hormone-replacement therapy or oral contraceptives
  - Tyrosine kinase inhibitor(s) (TKIs) approved by local regulatory authorities for treatment of cancer that have been discontinued > 7 days prior to Day 1 of Cycle 1; baseline scans must be obtained after discontinuation of prior TKIs, and criteria pertaining to adverse events attributed to prior cancer therapies must be met
  - Herbal therapy > 1 week prior to Day 1 of Cycle 1
  - Palliative radiotherapy for painful metastases or metastases in potentially sensitive locations (eg epidural space) > 2 weeks prior to Day 1 of Cycle 1
  - Prior anti-T cell immunoreceptor with immunoglobulin and immunoreceptor ITIM domains (TIGIT) agents are not allowed
- Prior treatment with cancer vaccines and/or cytokines is allowed provided that at least 6 weeks or five drug-elimination half-lives of the drug, whichever is shorter, have elapsed between the last dose and the proposed Day 1 of Cycle 1

- Any history of an immune-mediated grade 4 adverse event attributed to prior CIT (other than endocrinopathy managed with replacement therapy or asymptomatic elevation of serum amylase or lipase)

Treatment-specific exclusion criteria

- Active or history of autoimmune disease, including, but not limited to, systemic lupus erythematosus, rheumatoid arthritis, inflammatory bowel disease, vascular thrombosis associated with anti-phospholipid antibody syndrome, Wegener granulomatosis, Sjögren syndrome, Guillain-Barré syndrome, or multiple sclerosis with the following exceptions:
  - Patients with a history of autoimmune-related hypothyroidism who are on thyroid-replacement hormone are eligible for the study
  - Patients with eczema, psoriasis, lichen simplex chronicus, or vitiligo with dermatologic manifestations only (eg patients with psoriatic arthritis are excluded) are eligible for the study provided all the following conditions are met:
    - Rash must cover less than 10% of the body surface area
    - Disease is well controlled at baseline and only requires low potency topical steroids
    - No occurrence of acute exacerbations of the underlying condition requiring psoralen plus ultraviolet A radiation, methotrexate, retinoids, biologic agents, oral calcineurin inhibitors, or high-potency or oral corticosteroids within the previous 12 months
- Treatment with systemic immunosuppressive medication (including, but not limited to, corticosteroids, cyclophosphamide, azathioprine, methotrexate, thalidomide, and tumor necrosis factor-α [TNF-α] antagonists) within 2 weeks prior to Day 1 of Cycle 1
  - Patients who received acute, low-dose, systemic immunosuppressant medications are eligible for the study
  - Patients who received mineralocorticoids (eg fludrocortisone), inhaled corticosteroids for chronic obstructive pulmonary disease (COPD), or low-dose corticosteroids for adrenal insufficiency are eligible for the study
- History of idiopathic pulmonary fibrosis, pneumonitis (including drug induced), organizing pneumonia (i.e. bronchiolitis obliterans, cryptogenic organizing pneumonia, etc), or evidence of active pneumonitis on screening chest computed tomography (CT) scan
  - History of radiation pneumonitis in the radiation field (fibrosis) is permitted
- Active tuberculosis
- Severe infection within 4 weeks prior to Day 1 of Cycle 1, including, but not limited to, hospitalization for complications of infection, bacteremia, or severe pneumonia
- Recent infections not meeting the above criteria for severe infections, including the following:
  - Signs or symptoms of infection within 2 weeks prior to Day 1 of Cycle 1
  - Treatment with oral or intravenous antibiotics within 2 weeks prior to Day 1 of Cycle 1

    - Note: Patients receiving prophylactic antibiotics (eg for prevention of a urinary tract infection or COPD) are eligible

- Prior allogeneic bone marrow transplantation or solid organ transplantation
- Administration of a live, attenuated vaccine within 4 weeks before Day 1 of Cycle 1, or anticipation that such a live attenuated vaccine will be required during the study
- History of severe allergic, anaphylactic, or other hypersensitivity reactions to chimeric or humanized antibodies or fusion proteins
- Known hypersensitivity to Chinese hamster ovary (CHO) cell products
- Allergy or hypersensitivity to components of the atezolizumab formulation
