# Supplementary material for: Phase I pharmacokinetic, safety, and preliminary efficacy study of tiragolumab in combination with atezolizumab in Chinese patients with advanced solid tumors
Source: Cancer Chemother Pharmacol. 2024 Mar 7;94(1):45–55. doi: 10.1007/s00280-024-04650-y (PMC11258083; doi:10.1007/s00280-024-04650-y)
Supplement: Supplementary file 2 — Supplementary file2 (PDF 72 KB) [file 280_2024_4650_MOESM2_ESM.pdf]

Title: Phase I pharmacokinetic, safety, and preliminary efficacy study of tiragolumab in combination with atezolizumab in Chinese patients with advanced solid tumors

Authors: Dr. Colby S. Shemesh*, Prof. Yongsheng Wang*, Dr. Andrew An, Ms Hao Ding, Dr. Phyllis Chan, Ms Qi Liu, Dr. Yih-Wen Chen, Dr. Benjamin Wu, Dr. Qiong Wu, Prof. Xian Wang

*Co-first authors

Corresponding author: Colby S. Shemesh, Clinical Pharmacology, Genentech Inc., South San Francisco, CA, USA. E-mail: shemesh.colby@gene.com.

Journal: Cancer Chemotherapy and Pharmacology

Online resource 2 Treatment beyond progression criteria

- Due to the possibility of an initial increase in tumor burden caused by immune-cell infiltration in the setting of a T cell response (termed "pseudoprogression") with atezolizumab treatment, radiographic progression per Response Evaluation Criteria in Solid Tumors, version 1.1 (RECIST v1.1) may not be indicative of true disease progression
- In the absence of unacceptable toxicity, patients who meet criteria for disease progression per RECIST v1.1 while receiving atezolizumab will be permitted to continue atezolizumab if they meet all the following criteria:
  - Evidence of clinical benefit, as determined by the investigator following a review of all available data
  - Absence of symptoms and signs (including laboratory values, such as new or worsening hypercalcemia) indicating unequivocal progression of disease
  - Absence of decline in Eastern Cooperative Oncology Group performance score (ECOG PS) that can be attributed to disease progression
  - Absence of tumor progression at critical anatomical sites (eg leptomeningeal disease) that cannot be managed by protocol-allowed medical interventions
