# Supplementary material for: Phase I pharmacokinetic, safety, and preliminary efficacy study of tiragolumab in combination with atezolizumab in Chinese patients with advanced solid tumors
Source: Cancer Chemother Pharmacol. 2024 Mar 7;94(1):45–55. doi: 10.1007/s00280-024-04650-y (PMC11258083; doi:10.1007/s00280-024-04650-y)
Supplement: Supplementary file 3 — Supplementary file3 (PDF 64 KB) [file 280_2024_4650_MOESM3_ESM.pdf]

**Title: Phase I pharmacokinetic, safety, and preliminary efficacy study of tiragolumab in combination with atezolizumab in Chinese patients with advanced solid tumors**

**Authors: Dr. Colby S. Shemesh*, Prof. Yongsheng Wang*, Dr. Andrew An, Ms Hao Ding, Dr. Phyllis Chan, Ms Qi Liu, Dr. Yih-Wen Chen, Dr. Benjamin Wu, Dr. Qiong Wu, Prof. Xian Wang**

*Co-first authors

**Corresponding author:** Colby S. Shemesh, Clinical Pharmacology, Genentech Inc., South San Francisco, CA, USA. E-mail: shemesh.colby@gene.com.

**Journal:** Cancer Chemotherapy and Pharmacology

**Online resource 3** CONSORT diagram

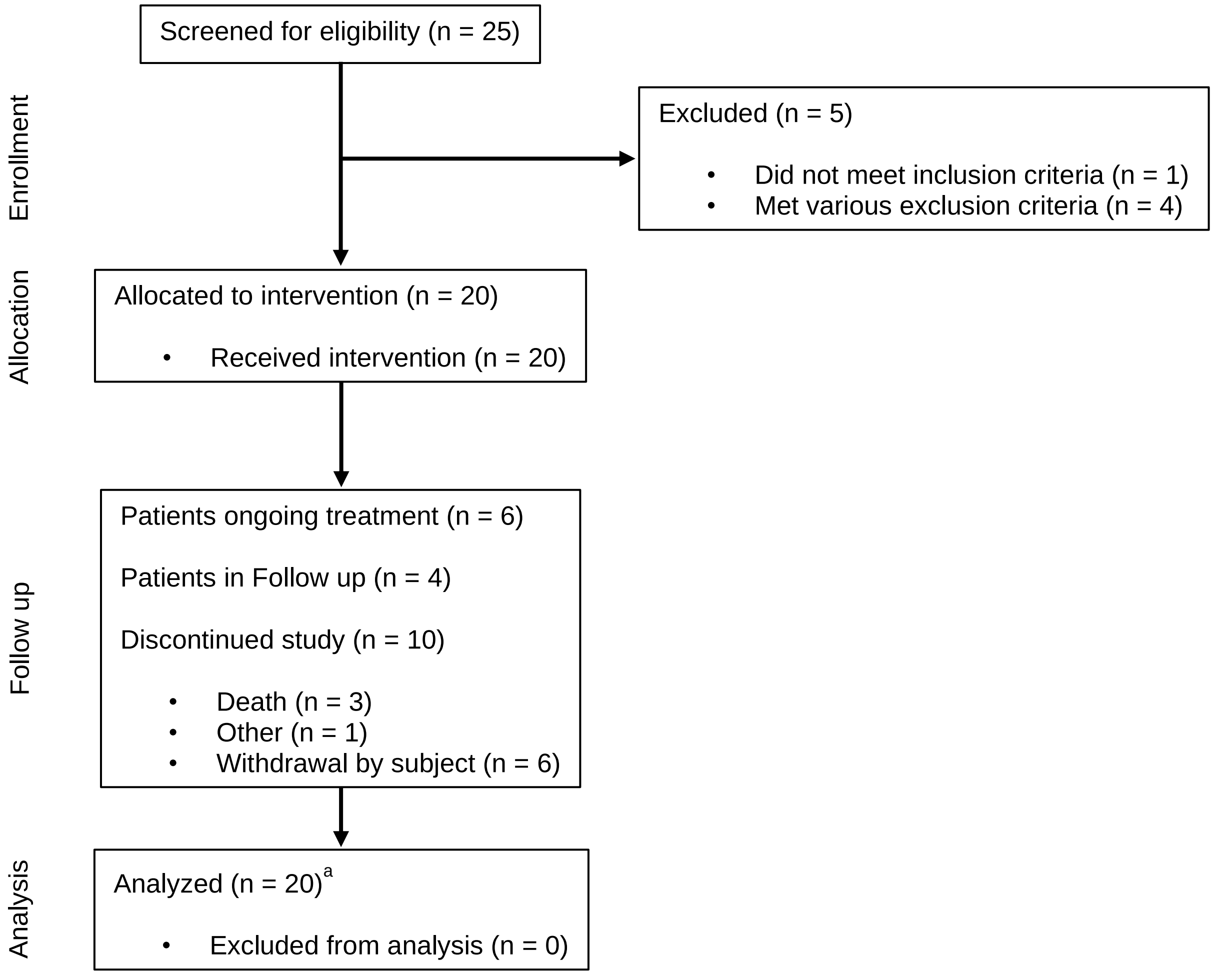


[a]All 20 patients at the data cut of date (10 February 2022) had completed protocol-specified treatment and blood sampling collection for adequate pharmacokinetic parameter estimation of relevant analytes during Cycle 1 to permit full characterization of tiragolumab and atezolizumab pharmacokinetics.
