# Supplementary material for: Phase I pharmacokinetic, safety, and preliminary efficacy study of tiragolumab in combination with atezolizumab in Chinese patients with advanced solid tumors
Source: Cancer Chemother Pharmacol. 2024 Mar 7;94(1):45–55. doi: 10.1007/s00280-024-04650-y (PMC11258083; doi:10.1007/s00280-024-04650-y)
Supplement: Supplementary file 4 — Supplementary file4 (PDF 69 KB) [file 280_2024_4650_MOESM4_ESM.pdf]

Title: Phase I pharmacokinetic, safety, and preliminary efficacy study of tiragolumab in combination with atezolizumab in Chinese patients with advanced solid tumors

Authors: Dr. Colby S. Shemesh*, Prof. Yongsheng Wang*, Dr. Andrew An, Ms Hao Ding, Dr. Phyllis Chan, Ms Qi Liu, Dr. Yih-Wen Chen, Dr. Benjamin Wu, Dr. Qiong Wu, Prof. Xian Wang

*Co-first authors

Corresponding author: Colby S. Shemesh, Clinical Pharmacology, Genentech Inc., South San Francisco, CA, USA. E-mail: shemesh.colby@gene.com.

Journal: Cancer Chemotherapy and Pharmacology

Online resource 4 Summary of common (≥ 10%) adverse events by preferred term, safety population from the YP42514 study

MedDRA preferred term	Tiragolumab plus atezolizumab, *N* = 20, (%)
**Total number of patients with at least one adverse event**	20 (100)
Total number of events	226
Aspartate aminotransferase increased	8 (40.0)
Rash	8 (40.0)
Lymphocyte count decreased	7 (35.0)
Pruritus	7 (35.0)
Alanine aminotransferase increased	6 (30.0)
Anemia	5 (25.0)
Gamma-glutamyl transferase increased	5 (25.0)
Hypertriglyceridemia	5 (25.0)
Hypokalemia	5 (25.0)
Blood alkaline phosphatase increased	4 (20.0)
Cough	4 (20.0)
Hypercholesterolemia	4 (20.0)
Platelet count decreased	4 (20.0)
Proteinuria	4 (20.0)
Alpha hydroxybutyrate dehydrogenase increased	3 (15.0)
Bilirubin conjugated increased	3 (15.0)
Blood bilirubin increased	3 (15.0)
Blood creatine phosphokinase increased	3 (15.0)
Blood fibrinogen increased	3 (15.0)
Hyperglycemia	3 (15.0)
Hyperuricemia	3 (15.0)
Hypocalcemia	3 (15.0)
White blood cell count decreased	3 (15.0)
Arthropathy	2 (10.0)
Back pain	2 (10.0)
Blood ketone body increased	2 (10.0)
Blood lactate dehydrogenase increased	2 (10.0)
Chest discomfort	2 (10.0)
Decreased appetite	2 (10.0)
Fatigue	2 (10.0)
Fibrin D dimer increased	2 (10.0)
Hemoptysis	2 (10.0)
Hypoalbuminemia	2 (10.0)
Neutrophil count decreased	2 (10.0)
Pleural effusion	2 (10.0)
Pyrexia	2 (10.0)
Sinus tachycardia	2 (10.0)
Skin lesion	2 (10.0)
Vertigo	2 (10.0)
Weight decreased	2 (10.0)

MedDRA, Medical Dictionary for Regulatory Activities.
