# Supplementary material for: Phase I pharmacokinetic, safety, and preliminary efficacy study of tiragolumab in combination with atezolizumab in Chinese patients with advanced solid tumors
Source: Cancer Chemother Pharmacol. 2024 Mar 7;94(1):45–55. doi: 10.1007/s00280-024-04650-y (PMC11258083; doi:10.1007/s00280-024-04650-y)
Supplement: Supplementary file 5 — Supplementary file5 (PDF 26 KB) [file 280_2024_4650_MOESM5_ESM.pdf]

Title: Phase I pharmacokinetic, safety, and preliminary efficacy study of tiragolumab in combination with atezolizumab in Chinese patients with advanced solid tumors

Authors: Dr. Colby S. Shemesh*, Prof. Yongsheng Wang*, Dr. Andrew An, Ms Hao Ding, Dr. Phyllis Chan, Ms Qi Liu, Dr. Yih-Wen Chen, Dr. Benjamin Wu, Dr. Qiong Wu, Prof. Xian Wang

*Co-first authors

Corresponding author: Colby S. Shemesh, Clinical Pharmacology, Genentech Inc., South San Francisco, CA, USA. E-mail: shemesh.colby@gene.com.

Journal: Cancer Chemotherapy and Pharmacology

Online Resource 5 Overview of AEs of special interest, safety population from the YP42514 study

	**Tiragolumab plus atezolizumab** ***N* = 20, (%)**
**Total number of patients with at least one AESI**	16 (80.0)
**Total number of AESI events**	46
**Total number of patients with at least one treatment-related AESI**	
Any treatment	13 (65.0)
Tiragolumab	12 (60.0)
Atezolizumab	13 (65.0)
**Grade 3-4 AESI**	2 (10.0)
Related to any treatment	2 (10.0)
**Grade 5 AESI**	0
Related to any treatment	0
**Serious AESI**	2 (10.0)
Related to any treatment	1 (5.0)
**AESI leading to withdrawal from treatment**	
Any treatment	1 (5.0)
Tiragolumab	1 (5.0)
Atezolizumab	1 (5.0)
**AESI leading to any dose interruption**	
Any treatment	1 (5.0)
Tiragolumab	1 (5.0)
Atezolizumab	1 (5.0)
**AESI medical concepts: patients with at least one**	
Immune-mediated hepatitis (diagnosis and abnormalities)	12 (60.0)
Immune-mediated hepatitis (lab abnormalities)	12 (60.0)
Immune-mediated rash	8 (40.0)
Immune-mediated hypothyroidism	2 (10.0)
Immune-mediated colitis	1 (5.0)
Immune-mediated hepatitis (diagnosis)	1 (5.0)
Immune-mediated pneumonitis	1 (5.0)
Infusion-related reactions	1 (5.0)
Autoimmune hemolytic anemia	0
Hemophagocytic lymphohistiocytosis	0
Immune-mediated adrenal insufficiency	0
Immune-mediated diabetes mellitus	0
Immune-mediated encephalitis	0
Immune-mediated Guillain-Barre syndrome	0
Immune-mediated hyperthyroidism	0
Immune-mediated hypophysitis	0
Immune-mediated meningitis	0
Immune-mediated meningoencephalitis	0
Immune-mediated myasthenia gravis	0

Immune-mediated myocarditis	0
Immune-mediated myositis	0
Immune-mediated myositis (myositis + rhabdomyolysis)	0
Immune-mediated nephritis	0
Immune-mediated ocular inflammatory toxicity	0
Immune-mediated pancreatitis	0
Immune-mediated severe cutaneous reactions	0
Immune-mediated vasculitis	0
Rhabdomyolysis	0

AE, adverse event; AESI, adverse events of special interest.
